# Anti-Apoptotic and Anti-Oxidant Proteins in Glioblastomas: Immunohistochemical Expression of Beclin and DJ-1 and Its Correlation with Prognosis

**DOI:** 10.3390/ijms20164066

**Published:** 2019-08-20

**Authors:** Elia Guadagno, Giorgio Borrelli, Sara Pignatiello, Annalidia Donato, Ivan Presta, Biagio Arcidiacono, Natalia Malara, Domenico Solari, Teresa Somma, Paolo Cappabianca, Giuseppe Donato, Marialaura Del Basso De Caro

**Affiliations:** 1 Department of Advanced Biomedical Sciences, Pathology Section, Federico II University of Naples, 80131 Napoli, Italy; 2 Department of Medical and Surgical Sciences–University of Catanzaro “Magna Graecia”–viale Europa, 88100 Catanzaro, Italy; 3 Department of Health Sciences, University of Catanzaro “Magna Græcia”-viale Europa, 88100 Catanzaro, Italy; 4 Department of Clinical and Experimental Medicine–University of Catanzaro “Magna Graecia”–viale Europa, 88100 Catanzaro, Italy; 5 Department of Neurosciences and Reproductive and Odontostomatological Sciences, Division of Neurosurgery, Federico II University of Naples, 80131 Napoli, Italy

**Keywords:** cell apoptosis, autophagy, glioblastoma, IDH1

## Abstract

DJ-1 deglycase is a protein with anti-oxidative and anti-apoptotic properties and its role in oncogenesis is controversial. Indeed in primary breast cancer and non-small-cell lung carcinoma, its higher expression was shown in more aggressive tumors while in other neoplasms (e.g., pancreatic adenocarcinoma), higher expression was related to better prognosis. Beclin has a relevant role in autophagy and cellular death regulation, processes that are well known to be impaired in neoplastic cells. DJ-1 shows the ability to modulate signal transduction. It can modulate autophagy through many signaling pathways, a process that can mediate either cell survival or cell death depending on the circumstances. Previously, it has been suggested that the involvement of DJ-1 in autophagy regulation may play a role in tumorigenesis. The aim of our study was to investigate the link between DJ-1 and Beclin-1 in glioblastoma through the immunohistochemical expression of such proteins and to correlate the data obtained with prognosis. Protein expression was assessed by immunohistochemistry and the immunoscores were correlated with clinicopathologic parameters. Kaplan–Meier survival curves were generated. A statistically significant association between DJ-1 score and recurrence (*p* = 0.0189) and between the former and Isocitrate Dehydrogenase 1 (IDH1) mutation (*p* = 0.0072) was observed. Kaplan–Meier survival curve analysis revealed that a higher DJ-1 score was associated with longer overall survival (*p* = 0.0253, ĸ2 = 5.005). Furthermore, an unexpected direct correlation (*p* = 0.0424, *r* = 0.4009) between DJ-1 and Beclin score was evident. The most significant result of the present study was the evidence of high DJ-1 expression in IDH-mutant tumors and in cases with longer overall survival. This finding could aid, together with IDH1, in the identification of glioblastomas with better prognosis.

## 1. Introduction

Glioblastoma represents the most common form of brain tumor among adults (15% of all intracranial neoplasms and 45% of primitive brain tumors) [1]. In 2016, the World Health Organization drafted a new classification system for brain tumors [2] that was based on the integration of morphological and molecular data. Glioblastoma is the most aggressive form (grade IV) of glioma and its morphology consists of the coexistence of four variables: nuclear atypia, mitoses, microvascular proliferation and necrosis.

Two main families of glioblastoma were identified, Isocitrate Dehydrogenase (IDH)-mutant and IDH-wild type glioblastomas, which represent two different clinicopathological diseases, the histopathology of which can be absolutely indistinguishable. IDH-wild type glioblastomas are the most frequent (90%). They are usually diagnosed at a median age of 62 years, with a median overall survival of 15 months after therapy (based on surgery plus chemotherapy and radiotherapy). In IDH-mutant glioblastomas, the median age at diagnosis is 44 years and with a median overall survival of 31 months after therapy. Other molecular alterations (p53, ATRX, TERT promoter mutations, EGFR amplification and mutations, etc.) can be detected in these tumors but not one is specific of each subtype.

DJ-1 deglycase (also called PARK7, Parkinson’s disease protein 7) is a protein with anti-oxidative and anti-apoptotic properties [3,4]. Under oxidative conditions, DJ-1 inhibits α-sinuclein aggregation through its chaperon activity, working as an oxidative stress sensor. Its oncogenic role was identified, for the first time, as a repressor of Phoshphatase and tensin Homolog (PTEN), inducing proliferation in primary breast cancer and primary non-small-cell lung carcinoma samples. It was observed [5,6,7,8] that a higher expression of DJ-1 was more common among cases with worse prognosis. Conversely, in cases of pancreatic and endometrial adenocarcinomas [9,10], the higher expression of the protein was related to a good prognosis.

Beclin is a protein with an essential role in autophagy and cellular death regulation [11], hence in tumorigenesis and in neurodegeneration. Furthermore, the caspase-mediated cleavage of Beclin 1 promotes crosstalk between apoptosis and autophagy [12]. Schizophrenia is linked to low levels of this protein in hippocampal tissue. Autophagy is a process that is preserved in evolution and it consists of the degradation of structural proteins and organelles. Its relationship with cancer is well known: in neoplastic cells, this process is impaired. The monoallelic deletion of the *Beclin* gene is observed in 40–75% of cases of sporadic breast cancer [13], and its heterozygous loss is associated with a higher proliferative rate and lower autophagy. These findings are indicative of a role for Beclin as a tumor suppressor [14,15]. It was observed that in those ovarian cancers [16] where autophagy was hyper-regulated, the lesion was less aggressive and more chemosensitive.

DJ-1 has the ability to modulate signal transduction. It can modulate autophagy through many signaling pathways, a process that can mediate either cell survival or cell death depending on the circumstances [17]. Previously, it has been suggested that the involvement of DJ-1 in autophagy regulation may play a role in tumorigenesis [18]. In particular, the overexpression of DJ-1 may inhibit Beclin 1 transcription.

The expression of DJ-1 in glioblastomas was studied, for the first time, in a series of 40 cases [19]. The study showed a direct correlation between the protein and p53, and an inverse association with EGFR. Furthermore, it was observed that DJ-1 was expressed more in astrocytes than in neurons, especially in reactive astrocytes, both in acute (e.g., infarction) and chronic (e.g., Parkinson’s disease) forms. In vitro experiments [20] suggest that its low expression makes cells more vulnerable. This observation is in favour of a possible role for this protein in promoting survival. In glioblastomas, DJ-1 positively modulates anti-apoptotic processes through two relevant pathways: one involving p53 and one through tyrosine kinases (EGFR, PI3K, Akt e PTEN). However, although this molecular link exists, the over-expression of DJ-1 is not effective enough to induce neoplastic transformation, unless there is co-transfection with another factor (e.g., Ras). Furthermore, astrocytes that chronically overexpress DJ-1 do not develop neoplasms, neither were any genetic mutations of DJ-1 detected in glioblastoma cases in studies on sequencing. In a case series of 76 ependymomas [21], the higher expression of the protein was observed in cases with a worse prognosis, as well as in higher-grade tumors in a study of 88 gliomas [22].

Most of the knowledge concerning the role of Beclin in gliomas is based on cell cultures. One study [23] evaluated the immunohistochemical expression of the protein and showed higher levels in tumor tissue compared to normal brain tissue, independently from tumor grade and overall survival.

The aim of our study was to investigate the link between DJ-1 and Beclin-1 in glioblastoma through the immunohistochemical expression of such proteins and to correlate the data obtained with prognosis.

## 2. Results

Of the 26 examined cases (Table 1), 17 were male and nine were female, aged between 35 and 81 years (median age of 63 years). All the patients were affected by a glioblastoma that was “localized” in the temporal, frontal and parietal lobe in 13, four and two cases, respectively, and “extended” in the fronto-temporal, tempo-parietal, tempo-parietal-occipital and parieto-occipital lobe in two, three, one and one cases, respectively. Three cases (cases 24, 25 and 26) were excluded from survival studies, as there was not enough follow-up time, since the patients are still alive and the surgery was performed less than 6 months ago. Overall survival was between 1 and 84 months. Disease recurrence was recorded in 16 of the 23 cases, with disease-free survival time ranging between 1 and 47 months.

A total of eight cases showed cytoplasmic reactivity to the IDH1 protein, a finding that was indicative of the presence of point mutation R132H in the *IDH1* gene.

**Figure 1:**
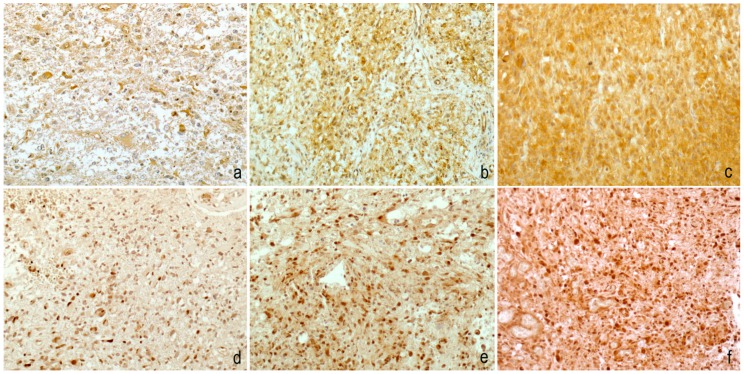
Immunohistochemical staining was evaluated with a four-tiered scoring system for both DJ-1 (**a**–**c**) and Beclin (**d**–**f**). DJ-1 nuclear and cytoplasmic/nuclear Beclin reactivity was observed in <10% of neoplastic cells in cases with a score of 1, in 10–50% of neoplastic cells in cases with a score of 2 and in ≥50% of neoplastic cells in cases with a score of 3. Score 0 was used in the absence of staining (200× magnification).

In all glioblastomas, the DJ-1 protein was expressed (Figure 1a–c) with a nuclear and cytoplasmic variable signal in 21 cases (9, 5 and 7 cases, respectively showed signal 1, 2 and 3 score).

**Figure 2:**
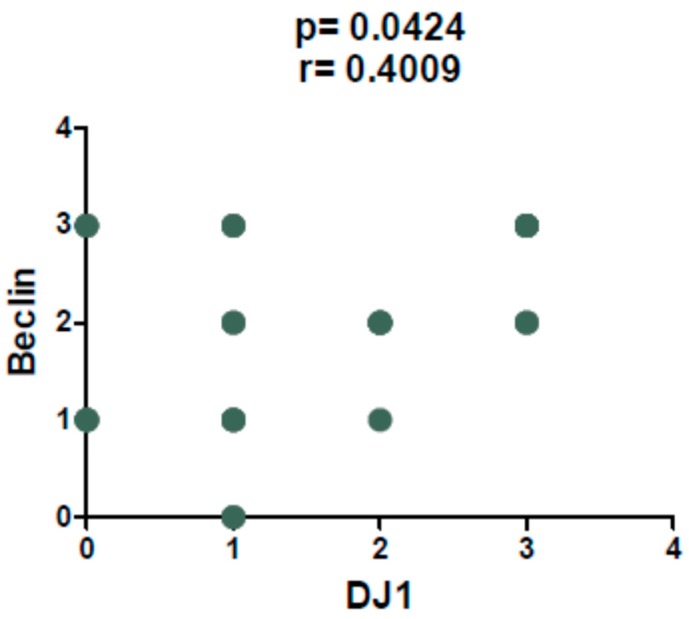
Spearman’s test revealed a direct correlation between DJ-1 and Beclin immunoscore (*p =* 0.0424, *r =* 0.4009).

The Beclin protein (Figure 1d–f) was negative in two cases and showed a signal with a score of 1 in seven cases, a score of 2 in eight cases and a score of 3 in nine cases. Spearman’s test highlighted a direct correlation between DJ-1 and Beclin score that was statistically significant (*p* = 0.0424, *r* = 0.4009) (Figure 2).

**Figure 3:**
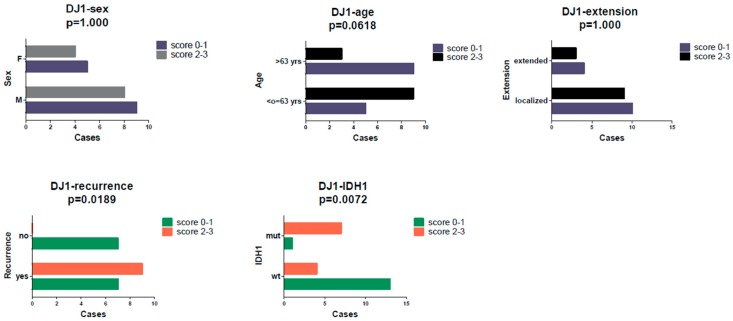
A high DJ-1 immunoscore (2–3) was associated with a higher recurrence rate (*p* = 0.0189) and IDH mutation.

**Figure 4:**
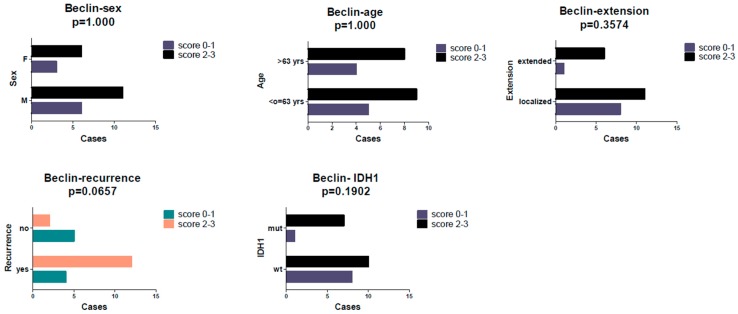
No statistically significant associations were found between Beclin immunoscore and clinicopathological features, but a slight tendency for a higher score in recurrent cases was detected (*p* = 0.0657).

Fisher’s exact test revealed a statistically significant association (Figure 3) between DJ-1 score (0/1 vs. 2/3) and recurrence (*p* = 0.0189) and between the former and IDH1 mutation (*p* = 0.0072). Furthermore, a slight tendency (Figure 4) to a more frequent relapse occurrence was observed in cases with a higher Beclin score (scores 2/3), without reaching statistical significance (*p* = 0.0657).

**Figure 5:**
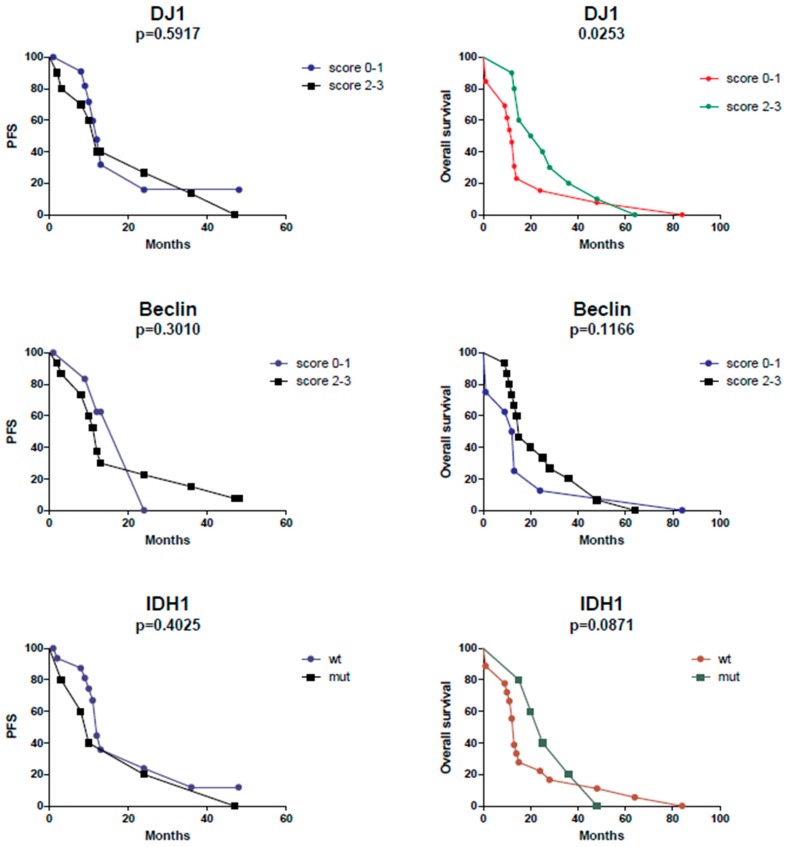
Cases with a DJ-1 score of 2 and 3 and cases showing IDH mutation showed longer overall survival. No statistically significant differences were evident in terms of progression-free survival (PFS).

The Kaplan–Meier curves analysis showed a statistically significant difference (*p* = 0.0253, ĸ2 = 5.005) in terms of overall survival in cases with a high (score of 2/3) compared to those with a low (score of 0/1) DJ-1 reactivity: a higher score was associated with longer overall survival (Figure 5). No effect on overall survival was recorded for Beclin.

Furthermore, in accordance with literature data, in our small collective, it was confirmed that cases with *IDH1* gene mutation have longer overall survival than *IDH* wild-type forms, although statistical significance was not reached (*p* = 0.0871, ĸ2 = 2.928).

No differences in terms of progression-free survival (PFS) were recorded for IDH1, DJ-1, or Beclin.

## 3. Discussion

Glioblastomas are highly aggressive high-grade neoplasms for which, to date, not many therapeutic options are available. The new WHO Classification system identified two major subpopulations that differ in terms of the presence of *IDH* gene mutation and overall survival. IDH- mutated forms have longer overall survival and represent the minority (approximately 10%) in the context of a rare neoplasm. Therefore, IDH mutation identification in glioblastoma is not of much relevance clinically. For this reason, many research lines are underway and they are aimed toward the identification of further prognostic factors and, eventually, predictive factors for personalized therapies.

DJ-1 is, by definition, a protein with anti-oxidant properties, that can preserve cancer cell survival. Hence, an oncogenic role should be the most possible. The relationship of DJ-1 protein with cancer is controversial: in some sites, its higher expression is associated with worse prognosis while in others, completely opposite findings were detected.

As observed by others, DJ-1 was more and variably expressed in glioblastomas compared to normal tissue. Furthermore, the protein showed a direct correlation with IDH1 expression and, consistently, with longer overall survival to disease. In glioblastoma, its clinical application could support IDH1 in achieving a prognostic stratification. IDH is an enzyme that catalyzes the oxidative decarboxylation of isocitrate, producing alpha-ketoglutarate (α-ketoglutarate) and CO2, an irreversible step of the citric acid cycle, due to its large negative free energy change. For this reason, it must be carefully regulated to avoid the unnecessary depletion of isocitrate (and therefore an accumulation of alpha-ketoglutarate). Furthermore, the mutated IDH1 protein acquires the ability to convert α-ketoglutarate to (R)-2-hydroxyglutarate (2-HG) which could act as an oncometabolite and DJ-1 could exert a potential protective role against oxidative stress in the tumor cells potentially triggered by 2-HG. The correlation between DJ-1 and the IDH-1 mutated form might be related to the activity of abnormal products of the metabolism of cancer cells since 2-HG contributes to metabolic reprogramming in diffuse glioma and hence to oxidative stress tolerance.

In view of the high levels of the protein found in pancreatic juices in cases of pancreatic adenocarcinoma [24], a potential role of circulating marker in the liquor or serum could be hypothesized. It could be detected during post-surgical follow up, for an early identification of relapse by a less invasive technique. Indeed, in our small series, DJ-1 was expressed more in cases where recurrence was more frequent.

The results obtained for Beclin were in accordance with what is known in the literature: the protein was expressed more in tumor tissue compared to normal tissue, but no statistically significant association with clinicopathological features was observed.

The only significant data was the direct correlation between DJ-1 and Beclin, an apparently unusual association that remains unclear, since the former is an anti-apoptotic protein and the other mainly performs an autophagic function. However, it is known that Beclin contains a Bcl2-homology-3 (BH3) domain and, in general, the “BH3-only members” of Bcl-2 family can bind to and antagonize the pro-survival proteins, leading to increased apoptosis (12). Beclin’s anti-apoptotic role is described in several settings including TNF-related apoptosis-inducing ligand (TRAIL), chemotherapy, irradiation, immunotherapy, nutrient deprivation, angiogenesis inhibitors and hypoxia. Probably, the overexpression of other factors, like High Mobility Group A (HMGA) which are general architectural chromatin proteins, that may induce autophagy can influence both the beclin-1 and DJ-1 levels in glioblastoma [25,26]. In an attempt to clarify the potential role of High-mobility group protein (HMGA1) factor on the regulation of DJ-1 gene expression, we performed a search using MatInspector software that looks for putative binding sites in DNA sequences. A total of 2000 bases upstream of the ATG codon of human DJ-1 gene was analyzed. The analysis revealed several sequence-specific bindings of the HMGA1 protein in a region between −2238 and −1563 bp upstream of the ATG site. Further, in vitro experiments are needed to validate the functional action of HMGA1 on the regulation of DJ-1 expression.

The microenvironment in which these factors operate could be decisive and could account for their different actions in different tumor types. Glioblastomas are tumors with different biological behaviors compared to purely epithelial tumors: the former usually show extreme local aggressiveness and very low metastatic capacity.

## 4. Materials and Methods

A total of 26 cases of glioblastomas were retrieved from the archives of the Institute of Pathology of Federico II University Hospital of Naples. They were diagnosed in the period ranging from 2004 to 2019, according to the current WHO Classification of tumours of the Central Nervous System [2]. In 23 cases, information concerning the clinical evolution (overall survival, relapse and, eventually, time to relapse) of the disease was available. A signed informed consent to use surgical specimens for scientific purposes was available for each patient.

All the specimens were formalin fixed and paraffin embedded. The most representative sample of the tumor was chosen to perform immunohistochemical studies.

After deparaffinization, slides were submerged in either sodium citrate buffer or Tris–EDTA buffer for heat-induced epitope retrieval at 97 °C for 20 min. Immunohistochemistry for DJ-1 and Beclin proteins was performed using the Polyclonal Rabbit Anti-Human *DJ-1* antibody (NovusBiologicals; 1: 800 dilution) and Polyclonal Rabbit Anti-Human *Beclin* antibody (NovusBiologicals; 1: 1000 dilution). Brain tissue with reactive astrocytes and adrenal medulla were chosen as a positive control, respectively for DJ-1 and Beclin. Sections of glioblastoma stained with the secondary antibody alone were used as a negative control.

For DJ-1 (nuclear and cytoplasmic signal) and Beclin (nuclear signal), immunoreactivity was assessed on 10 power fields with a semiquantitative score on neoplastic cells: 0 (no staining), 1 (<10% of cells), 2 (10−50%) and 3 (≥50%) (Figure 1). Scores 2 and 3 were considered high. Each case was evaluated independently by three different observers and when the scores were discordant, the cases were re-evaluated with a multi-head microscope. In all cases, IDH1 staining was automatically made with pre-diluted antibody (dia-h09, Dianova, 1: 20 dilution).

IDH status was assessed by immunohistochemistry in most cases but in patients under the age of 54, it was also necessary to carry out molecular investigations to exclude non-canonical IDH mutations not detectable with the antibody (IDH1 R132H). Indeed, it is well known [2] that in patients aged >54 years, the probability of having an alternative IDH mutation is <1%. Therefore, immunohistochemistry alone may be sufficient to investigate IDH status.

For correlation studies between scores, Spearman’s test was applied, and Fisher’s exact test was also performed for studies on the association with clinicopathological parameters (sex, age, tumor extension, relapse and IDH status).

Kaplan–Meier survival curves for DJ-1, Beclin and IDH1 were generated with the Gehan–Breslow–Wilcoxon test.

All statistical analyses were performed using GraphPad Prism 5 software (GraphPad Software, La Jolla, CA, USA). A probability (*p*) value less than 0.05 was considered statistically significant.

## 5. Conclusions

Overall, the present study provides preliminary data for the evaluation of anti-apoptotic and anti-oxidant proteins in gliomas. As already observed in other studies, DJ-1 was variably expressed in glioblastomas. In our study, it was shown that its higher expression was related to longer overall survival and to the IDH-mutant state. This finding could support the identification of cases with better prognosis. It was also shown that Beclin was variably expressed in the same study but, in this case, no correlation with clinicopathological parameters was detected. Furthermore, un unexpected direct correlation between Beclin and DJ-1 was observed, a finding deserving further studies on cell cultures and on a larger scale.

## Figures and Tables

**Table 1:** Clinico-patohologic features of the collective.

Case	Sex	Age	Site	IDH1	DJ-1	Beclin	Recurrence	O.S.*
1	F	62	Right temporal	wt	1	0	24	84
2	F	70	Right temporal	wt	1	3	no	10
3	M	35	Left temporal	mut	2	2	47	48
4	F	41	Right frontal	mut	2	2	24	36
5	F	58	Left posterior frontal	wt	2	2	2	12
6	M	74	Left temporal	wt	3	3	12	15
7	M	60	Left temporale	wt	0	1	no	12
8	M	57	Right frontal-temporal	wt	0	3	no	48
9	F	36	Left posterior temporal	wt	1	1	12	24
10	M	69	Right temporal	wt	1	0	9	13
11	F	72	Left temporal	wt	3	2	36	64
12	M	73	Left posterior parietal	wt	1	2	10	11
13	M	71	Left parietal	wt	1	2	8	9
14	M	41	Right temporal	mut	3	3	8	15
15	M	52	Left temporal	mut	3	3	3	20
16	M	63	Left frontal	mut	3	3	10	25
17	M	61	Left temporal-parietal	wt	2	2	12	28
18	M	69	Right frontal-temporal	wt	1	3	13	13
19	F	81	Right parietal-occipital	wt	0	3	11	14
20	F	72	temporal-parietal-occipital	wt	1	1	no	1
21	M	62	Right temporal	wt	1	1	no	9
22	M	75	Right temporal	wt	0	1	no	1
23	M	63	Right frontal	wt	2	1	no	13
24	M	70	Right temporal-parietal	mut	3	3	n.a.	n.a.
25	M	72	Left temporale	mut	0	1	n.a.	n.a.
26	F	39	Left frontal-parietal	mut	3	2	n.e.	n.e.

OS*: overall survival; n.a.: not available because follow-up time was insufficient (<6 months) in patients still alive.
